# Olfactory ensheathing cells and neuropathic pain

**DOI:** 10.3389/fcell.2023.1147242

**Published:** 2023-04-05

**Authors:** Ji-peng Liu, Jia-ling Wang, Bai-er Hu, Fei-long Zou, Chang-lei Wu, Jie Shen, Wen-jun Zhang

**Affiliations:** ^1^ Department of Rehabilitation Medicine, The Second Affiliated Hospital, Nanchang University, Nanchang, Jiangxi, China; ^2^ Department of Gastrointestinal Surgery, The Second Affiliated Hospital, Nanchang University, Nanchang, Jiangxi, China; ^3^ Department of Physical Examination, The Second Affiliated Hospital, Nanchang University, Nanchang, Jiangxi, China

**Keywords:** OECs, nerve injury, neuropathic pain, treatment, cell transplantation

## Abstract

Damage to the nervous system can lead to functional impairment, including sensory and motor functions. Importantly, neuropathic pain (NPP) can be induced after nerve injury, which seriously affects the quality of life of patients. Therefore, the repair of nerve damage and the treatment of pain are particularly important. However, the current treatment of NPP is very weak, which promotes researchers to find new methods and directions for treatment. Recently, cell transplantation technology has received great attention and has become a hot spot for the treatment of nerve injury and pain. Olfactory ensheathing cells (OECs) are a kind of glial cells with the characteristics of lifelong survival in the nervous system and continuous division and renewal. They also secrete a variety of neurotrophic factors, bridge the fibers at both ends of the injured nerve, change the local injury microenvironment, and promote axon regeneration and other biological functions. Different studies have revealed that the transplantation of OECs can repair damaged nerves and exert analgesic effect. Some progress has been made in the effect of OECs transplantation in inhibiting NPP. Therefore, in this paper, we provided a comprehensive overview of the biology of OECs, described the possible pathogenesis of NPP. Moreover, we discussed on the therapeutic effect of OECs transplantation on central nervous system injury and NPP, and prospected some possible problems of OECs transplantation as pain treatment. To provide some valuable information for the treatment of pain by OECs transplantation in the future.

## 1 Introduction

Nervous system encounters noxious stimuli (such as inflammation, trauma, mechanical and immune), which can lead to different degrees of nerve damage and induce neuropathic pain (Sato et al., 2014; Philpott et al., 2017; Lovaglio et al., 2019). NPP is a kind of chronic pain, which can persist with a variety of diseases. It has the same manifestation with other types of chronic pain (cancer pain and inflammatory pain), mainly expressed as abnormal pain, hyperalgesia and spontaneous pain (Gierthmühlen and Baron, 2016; Finnerup et al., 2021). Although these pains can exist individually or simultaneously, there are certain differences in the expression and degree of pain due to individual differences. So, the diagnosis and treatment of NPP lack a unified standard, which brings great difficulties in treatment. The injury of central spinal cord and peripheral nerve is the key to induce pain. In addition, some other diseases, such as ischemic injury (Lin et al., 2018), tumor (Gül et al., 2020), inflammatory stimulation (Li et al., 2021a), can stimulate or damage nerves and induce pain in different degrees (Cohen and Mao, 2014). In view of this, it is particularly important to find treatments for pain relief. Currently, great progress has been made in the treatment of NPP, such as drug therapy (Lee et al., 2018a) and physiotherapy (Finnerup et al., 2015), but the effect is still not ideal, especially in drug therapy, which is not only short-term, but also has certain dependence and side effects. Therefore, exploring new and promising treatment methods is on the agenda.

In recent years, with the continuous research and exploration of nerve injury and pain treatment, the concept of cell transplantation has been introduced (Sullivan et al., 2016). Researchers have tried to transplant some special types of cells or intravenously into the host (such as spinal cord injury, sciatic nerve injury, and trigeminal nerve injury) to relieve pain and have achieved good progress (Chen et al., 2013; Ito et al., 2020). Indeed, cell transplantation technology has achieved remarkable results in the treatment of nerve injury. Such as neural stem/progenitor cells (Lee et al., 2021a), olfactory ensheathing cells (Guerout et al., 2014), adipose stem cells (Li et al., 2018), mesenchymal cells (Cofano et al., 2019) and Schwann cells (Li et al., 2020a) in the central nervous system (CNS) and peripheral nervous system (PNS) have neurorestorative effects (Assinck et al., 2017; Kubiak et al., 2020). For example, transplantation of mesenchymal stem cell (MSCs)-derived exosomes into locally injured nerve tissue can effectively reduce inflammation and oxidation, and significantly restore injured nerves (Li et al., 2020b). Accordingly, these cells with functional properties are gradually being used in the treatment of pain, including inflammation-induced pain and cancer-induced pain (Wei et al., 2019; Zhang et al., 2020a). These cells can continuously secrete neuroactive nutritional factors for analgesia (such as neurotrophin Y, brain-derived neurofactor, b-endorphin, enkephalin and vascular growth factor). It has a good effect on long-term analgesia (Chen et al., 2013). Indeed, these living cells transplanted into the body can play a role in alleviating hyperalgesia or lowering pain threshold in the host (Lindvall, 2015; He et al., 2019).

OECs are glial cells that have been studied more in recent years. They can exist for life in the central and peripheral nervous system, constantly renew and survive, secrete a variety of neurotrophic factors, reduce local inflammatory response (such as the release of TNF-a, IL-1β, and IL-18), change the local surrounding microenvironment, repair damaged nerves and reduce inflammatory reaction (Reshamwala et al., 2019; Gilmour et al., 2020). It is worth mentioning that in the treatment of other diseases [such as retinopathy (Yu et al., 2022), dorsal root injury (Minkelyte et al., 2021) and Parkinson’s disease (Weng et al., 2020)], OECs also have a good effect. Correspondingly, OECs have also achieved certain recognition and achievements in the treatment of NPP. Indeed, OECs transplantation contributes to pain-relieving effects (Zheng et al., 2017). Studies have shown that OECs transplantation significantly increases NF200 expression, reduces GFAP expression, improves sensory function after spinal cord injury, reduces P2X4R overexpression, and relieves NPP (Zheng et al., 2017). These findings identify an important contribution of OECs transplantation in the treatment of nerve injury and pain. Therefore, in this paper, we explored the efficacy of OECs on nerve injury repair and the treatment of NPP, and OECs are expected to become a new method and technology for the treatment of pain in the future.

## 2 Biological characteristics of OECs

Regeneration and repair of nervous system injury is a complex process involving multiple steps and factors. On the one hand, the key to nerve injury repair lies in the survival and function of neurons. On the other hand, damaged axons have the function of bridging the damaged site or broken end (Jessen and Mirsky, 2016; Min et al., 2021). Importantly, axonal regeneration requires remyelination and reconstitution of functional synapses, delivery of neurotrophic factors, improved inflammation, and modulation of immune responses, which are critical for nerve injury repair and regeneration (Otto, 2021). Different studies have found that transplanted cells, such as neural stem cells, have functional properties to accomplish these processes (Hu et al., 2019; Shao et al., 2019). Recently, a large number of studies have revealed a kind of glial cells-OECs, which can promote axonal regeneration and nerve tissue reconstruction, improve functional characteristics, and have received extensive attention in the field of tissue engineering and injury repair (Wright et al., 2018; Collins et al., 2019).

The role of OECs in nerve injury repair and regeneration has been confirmed by more and more studies. The biological characteristics and mechanisms of OECs in repairing damaged nerves and inhibiting pain have been recognized and affirmed, mainly in the following aspects (Table 1):

a OECs can survive and renew for life in the central nervous system, ensuring the number and vitality (Roet and Verhaagen, 2014; Li et al., 2021b).
b Abundant sources of OECs: In animal model studies, OECs are derived from the olfactory bulb tissue and olfactory mucosa (the most superficial layer and the lamina propria), which belong to the central nervous system and peripheral nervous system respectively (Guerout et al., 2014). The *in vitro* culture survival rate and purity are as high as 95% or more, which is also the key factor for experimental and clinical *in vitro* culture of OECs (Dombrowski et al., 2006; Delaviz et al., 2008; Radtke et al., 2009; Roet and Verhaagen, 2014).
c Heterogeneity of OECs: Primary OECs are easier to obtain from embryonic animals and early postnatal animals, but it is more difficult to obtain purified OECs due to the abundance of non-OECs in the embryonic and early postnatal olfactory bulbs and olfactory mucosa. Conversely, OECs can be readily purified and cultured from adult olfactory bulbs and olfactory mucosa. Moreover, OECs are obtained from different parts, and there are also certain differences in their effects on nerve damage repair. An important factor is that *in vitro* culturing of OECs (e.g., culture conditions and media) can affect OECs (Paviot et al., 2011; Honoré et al., 2012). In addition, OECs derived from the olfactory mucosa are closely related to neurons and fibroblasts, while OECs derived from the olfactory bulb are closely related to fibroblasts and astrocytes (Lakatos et al., 2000; Jani and Raisman, 2004; Windus et al., 2010). Compared with olfactory bulb-derived OECs, olfactory mucosa-derived OECs can promote nerve regeneration more (Li et al., 2020c). This means that OECs from different sources may have completely different ability to promote nerve regeneration and the efficacy after transplantation. Therefore, there is a certain heterogeneity in OECs from different sources.
d OECs have the function of secreting various neurotrophic factors, such as neurotrophin Y, brain-derived neurotrophic factor and VEGF: Transplantation of OECs has a strong potential to provide an adaptive microenvironment for damaged nerves, one of which is characterized by the secretion of trophic factors and cellular matrix, improving the microenvironment for axon regeneration and extension (Nazareth et al., 2020). Indeed, OECs can secrete platelet-derived growth factor, neuropeptide Y, glial-derived connexin, S100, ciliary neurotrophic factor (CNTF), these nutrients provide a nutritional basis for nerve regeneration and repair (Ye and Houle, 1997; Ahuja et al., 2020). Importantly, OECs can produce NT-3, and BDNF which effectively promote neuronal survival and axonal sprouting, even extending longer distances within areas of CNS injury (Ma et al., 2010; Gu et al., 2017).
e OECs secrete cell adhesion molecules: In addition to the production of trophic factors, another important feature of OECs is that OECs can produce a series of extracellular matrix proteins, which have been confirmed by different studies to promote neuronal survival, axon regeneration and extension, especially the function of axon guidance (Liu et al., 2010). Indeed, an important mechanism for OECs to promote nerve injury repair is through the secretion of adhesion molecules [such as L1-neural cell adhesion molecule (L1-NCAM) and neural cell adhesion molecule 1 (NCAM1)] (Witheford et al., 2013; Tang et al., 2017; Guo et al., 2020).
f OECs promote axon regeneration and inhibit glial scarring and cavitation (DeLucia et al., 2003; Khankan et al., 2015): Studies have shown that the transplantation of OECs into the injured part of the T8 spinal cord in rats can increase the axonal growth of the neurospinal tract, the blue pulp and the corticospinal tract at the stump of the spinal cord. The expression of GFAP and NG2 is down-regulated in the spinal cord segments around the injury, suggesting that there is a more suitable environment for axon regeneration (López-Vales et al., 2006). OECs treatment can increase the number of regenerated neurons, improve the morphology of nerve fibers, increase the number of myelinated nerve fibers, nerve fiber diameter and myelin sheath thickness (Gu et al., 2019).
g Another interesting feature is the phagocytic activity of OECs (Nazareth et al., 2021): In the past, it was thought that OECs and astrocytes had some similar characteristics, but later it was further found that OECs and microglia also had common characteristics, which means that OECs have the function of phagocytosis of bacteria (Vincent et al., 2007; Panni et al., 2013; Nazareth et al., 2015). OECs or Schwann cells (SCs) are thought to aid regeneration by removing necrotic cells (necrotic bodies, NBS) as well as myelin debris. Interesting finding is that OECs have higher phagocytosis and transport capacity than stem cells (Nazareth et al., 2020). OECs degrade neurocalcitonin, an inhibitor of axonal regeneration, by secreting matrix metalloproteinase-2, which promotes nerve regeneration (Yui et al., 2014). Effective removal of neurodegenerative and apoptotic nerve tissue fragments and cell products are essential for creating an environment that allows damaged neurons to regenerate. The phagocytic activity of OECs can make a substantial contribution to the growth of neurons in this harsh environment, which depends on the phenotypic changes of OECs (enhanced activation and phagocytosis), and this enhanced phagocytic activity greatly promotes the growth of neurons under hostile culture conditions (Hao et al., 2017).
h OECs have the function of “bridge” and remyelination formation ability (Franklin, 2003): Purified OECs express PSA-E-NCAM (polysialo neural cell adhesion molecule) and tenascin, which promote axonal elongation and remyelination ability (Higginson and Barnett, 2011).
i OECs can reduce the local inflammatory response of nerve injury (inhibit the pro-inflammatory effect of astrocytes), reduce the release of inflammatory factors (such as TNF-a), improve the microenvironment around local inflammation, and have a better role in repairing damaged nerves (Su et al., 2009; Chuah et al., 2011). The study found that OECs secreted αB-crystallin (CryAB), an anti-inflammatory protein that coordinated the immune response between cells, and also inhibited the production of neurotoxins by astrocytes, created an environment for nerve growth (Saglam et al., 2021).
j Migration ability of OECs (Santos-Silva et al., 2019): The lipid rafts of OECs are rich in cholesterol, sphingomyelin, phosphatidylcholine, caveolin-1, Flotillin-1, ganglioside Gm1 and gangliosides recognized by 9murine O-acetyl GD3, A2B5-recognized gangliosides, CNPase, α-Actinin and β-1-integrin (Campos et al., 2021). Analysis of the OECs actin cytoskeleton reveals stress fibers, membrane spinous processes, folded membranes and lamellar fat deposits during cell migration and the distribution of α-Actinin in membrane projections (Campos et al., 2021). For the first time, α-actin and Flotillin-1 were found in OECs lipid rafts, suggesting that membrane lipid rafts together with β-1-integrin and gangliosides play a role in the migration of OECs (Campos et al., 2021). OECs have migratory roles in both physiological and pathological conditions of the nervous system, such as inflammation, hypoxia, aging, neurodegenerative diseases, head trauma, brain tumors, and infections (Grassi et al., 2020).

**TABLE 1:** Biological characteristics of olfactory ensheathing cells (OECs).

● Lifelong survival and renewal within the nervous system
● Abundant sources of OECs
a. Olfactory bulb tissue
b. Olfactory mucosa (superficial and lamina propria)
● Heterogeneity of OECs: Different sources of OECs have differences in repairing damaged nerves
● Secretes various neurotrophic factors: Provide a good basic microenvironment for nerve regeneration
● Secreted cell adhesion molecule
● Inhibits glial scarring and void formation
● Phagocytic activity: Removal of necrotic cells (including neurons) and myelin debris
● Has a “bridge” effect, promoting axonal regeneration and remyelination
● Reduce neuroinflammatory response
● Migration ability

## 3 The role of OECs in treatment of nerve injury

It is understood that OECs are present in the olfactory bulb and olfactory mucosa, and exist and renew for life in the central and peripheral nervous system. Therefore, the role of OECs in the repair of central injury and peripheral nerve injury has been widely studied and recognized (Duan and Lu, 2015; Khankan et al., 2016). Indeed, OECs transplantation exerts its biological characteristics to improve the microenvironment at the injured nerve site and promote nerve repair. Nerve damage can lead to permanent loss of motor and sensory function, yet current treatments are limited and some are ineffective, inspiring researchers to explore new and promising treatment methods. Fortunately, OECs transplantation has emerged as a possible therapy for CNS or PNS injury or other neurological disorders in animal models (Goulart et al., 2016; Wang et al., 2021a; Lee et al., 2021b). This is because transplanting OECs into the damaged site of the damaged nerve can create better survival conditions for the damaged neurons.

**FIGURE 1:**
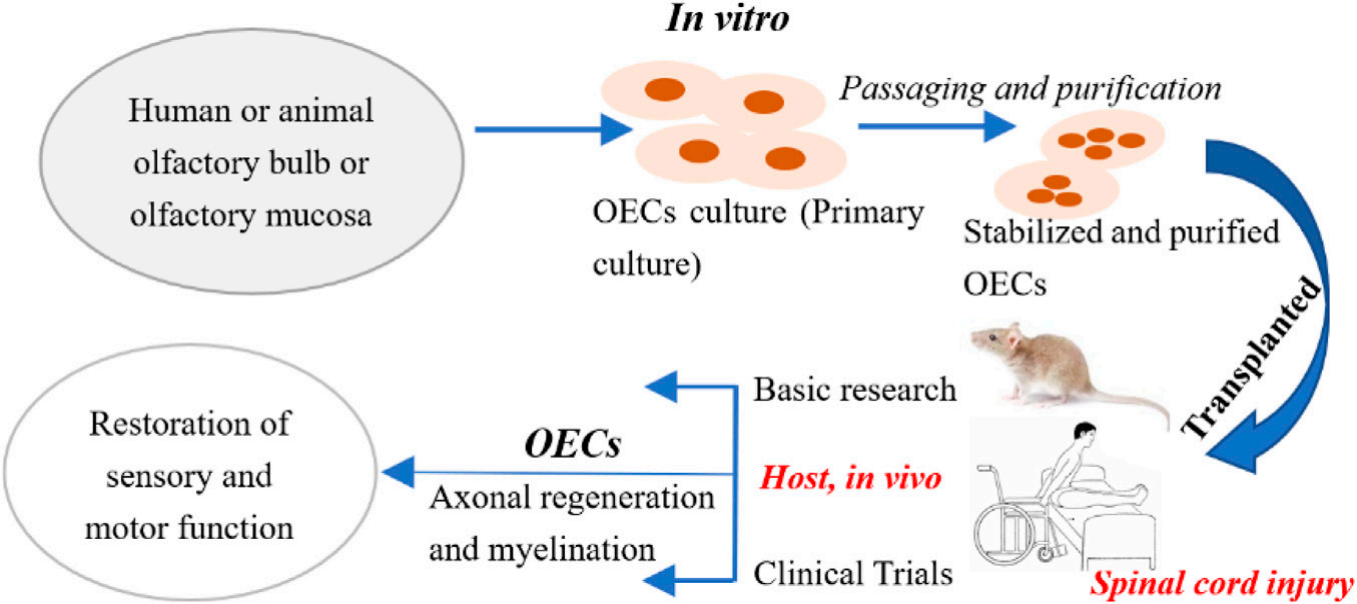
Procedure for OECs transplantation of the treatment for spinal cord injury. Step 1: Take olfactory bulb tissue or olfactory mucosa from animals or humans; Step 2: Pay attention to aseptic operation throughout the process, and *in vitro* primary culture OECs; Step 3: Purify and identify the cultured OECs to obtain stable OECs; Step 4: Transplant the stably cultured OECs into the host; Step 5: Check the host’s sensory and functional recovery after OECs transplantation.

Spinal cord injury is a common site in the central nervous system, and can lead to sensory and motor dysfunction after (Muniswami and Tharion, 2018; Voronova et al., 2018). OECs transplantation can promote axonal regeneration and myelination in spinal cord injury, and can restore part of sensory and motor functions, which all lie in the biological function of OECs (Reshamwala et al., 2019) (Figure 1). The transplantation of OECs to the injured spinal cord can limit the activation and penetration of immune cells, protect axons and neurons, reduce the secretion of inhibitory molecules in the lesion core, and exert neuroprotective and immunomodulatory effects (Khankan et al., 2016). It is worth mentioning that the transplantation of OECs has the effect of promoting the regeneration of the injured site of the blood vessels (Beiersdorfer et al., 2019; Beiersdorfer et al., 2020; Wang et al., 2022). For example, OECs can promote the increase of vascular endothelial growth factor-A and platelet-derived growth factor-AA, effectively promoting the proliferation, migration and formation of vascular-like structures of vascular endothelial cells (Wang et al., 2022).

Another important role is that OECs can inhibit the activation of microglial and reduce neuroinflammation (Zhang et al., 2019a; Guo et al., 2020). After OECs transplantation, the expression of interleukin-1 receptor antagonist (IL-1ra) was up-regulated, and the expression of many chemokines, including proinflammatory chemokine IL-1α and IL-1β was down-regulated, which inhibited the activation of microglia (Zhang et al., 2021). Other studies found that the combination of OECs and minocycline reduced cavitation and astrogliosis, further reduced levels of IL-1β, TNF-α, caspase-3 and oxidative stress (Pourkhodadad et al., 2019).

Moreover, scarring caused by astrocytes at the edge of spinal cord injury can hinder axonal regeneration (Deng et al., 2011; Hara et al., 2017; Okada et al., 2018). OECs can integrate and migrate with astrocytes at the site of spinal cord injury, providing a bridge for axons to penetrate scars and grow to the lesion core (Witheford et al., 2013; Khankan et al., 2015). Studies have been found that the combined transplantation of OECs and spinal cord decellularized scaffolds can better promote axonal regeneration, improve the proliferation and distribution of astrocytes at the injury site, and restore some functions (Yu et al., 2021). Another important finding is that OECs can associate with other cells (such as adipose stem cells, neural stem cells and mesenchymal stem cells) (Wang et al., 2010; Yazdani et al., 2012; Gomes et al., 2018), biomaterials (such as albumin scaffolds and spinal cord cell scaffolds) (Ferrero-Gutierrez et al., 2013; Yu et al., 2021) and physical therapy methods (such as exercise training and stimulation) (Thornton et al., 2018; Prager et al., 2021) play a better role in promoting the repair of spinal cord injury. It has been found that the combined transplantation of nerve stem cells (NSCs) and OECs has a synergistic effect on spinal cord injury and promotes nerve regeneration and functional reconstruction (Ao et al., 2007). They also found that OECs could bridge between nerve injuries while performing axon elongation and promoting myelination (Ao et al., 2007). Other studies have further found that co-transplantation of OECs and NSCs can promote the proliferation and differentiation of neural stem cells *in vitro*. Interestingly, OECs can improve the survival rate of NSCs *in vivo* and inhibit the programmed necrosis of NSCs after co-transplantation, thus enhancing the therapeutic effect of spinal cord injury (Wang et al., 2021b).

Successful results in animal models show that OECs transplantation is safe and reliable for the treatment of spinal cord injury. Correspondingly, some clinical trials of OECs transplantation for the treatment of spinal cord injury have also been carried out, and certain results have been achieved (Tabakow et al., 2014; Gomes et al., 2016; Gómez et al., 2018). Researchers transplanted human fetal OECs into the upper and lower ends of spinal cord injuries in 300 patients by intraspinal injection, it was found that 300 patients with complete SCI (ASIA grade A) and incomplete SCI (ASIA grade D), rapidly improved some neurological functions (Huang et al., 2006). *Wu et al* used fetal OECs in a clinical trial in patients with spinal cord injury and tested the safety of six patients with chest injuries and the efficacy of five patients with neck injuries. They found that OECs showed a good safety, with improvements in sensation and spasticity, but the treatment effect was suboptimal (Wu et al., 2012). Although the OECs obtained from the fetus have a certain effect in the treatment of spinal cord injury, some researchers believe that whether the cells obtained from the fetus are pure OECs is still controversial (Hummel et al., 2007). The investigators transplanted autologous OECs into the site of six patients with complete thoracic paralysis and followed up for up to 3 years. They did not find any tumorigenic cells, syringomyelia or other adverse radiological phenomena (Mackay-Sim et al., 2008). Strangely, most patients showed no significant functional changes, but in one patient, both tactile and acupuncture sensitivity were found to improve (Mackay-Sim et al., 2008). They believe that it is feasible and safe to transplant autologous OECs into the injured spinal cord.

Taken together, OECs show a good repair effect in basic experiments, although OECs transplantation also show a certain therapeutic effect in clinical trials, but the results of these clinical trials are not satisfactory. Whether OECs are effective in promoting neural repair after spinal cord injury remains unknown. Therefore, more research data are needed to support the application of OECs in clinical treatment. At present, there are some obstacles to the use of OECs in clinical trials, such as difficulties in obtaining autologous cells, adverse reactions, correct transplantation methods, cell usage and sources, identification and purification of human OECs, etc. Therefore, the use of OECs for the treatment of clinical patients in the future requires a large number of experiments to provide a solid foundation.

## 4 Pathological mechanism of neuropathic pain

**FIGURE 2:**
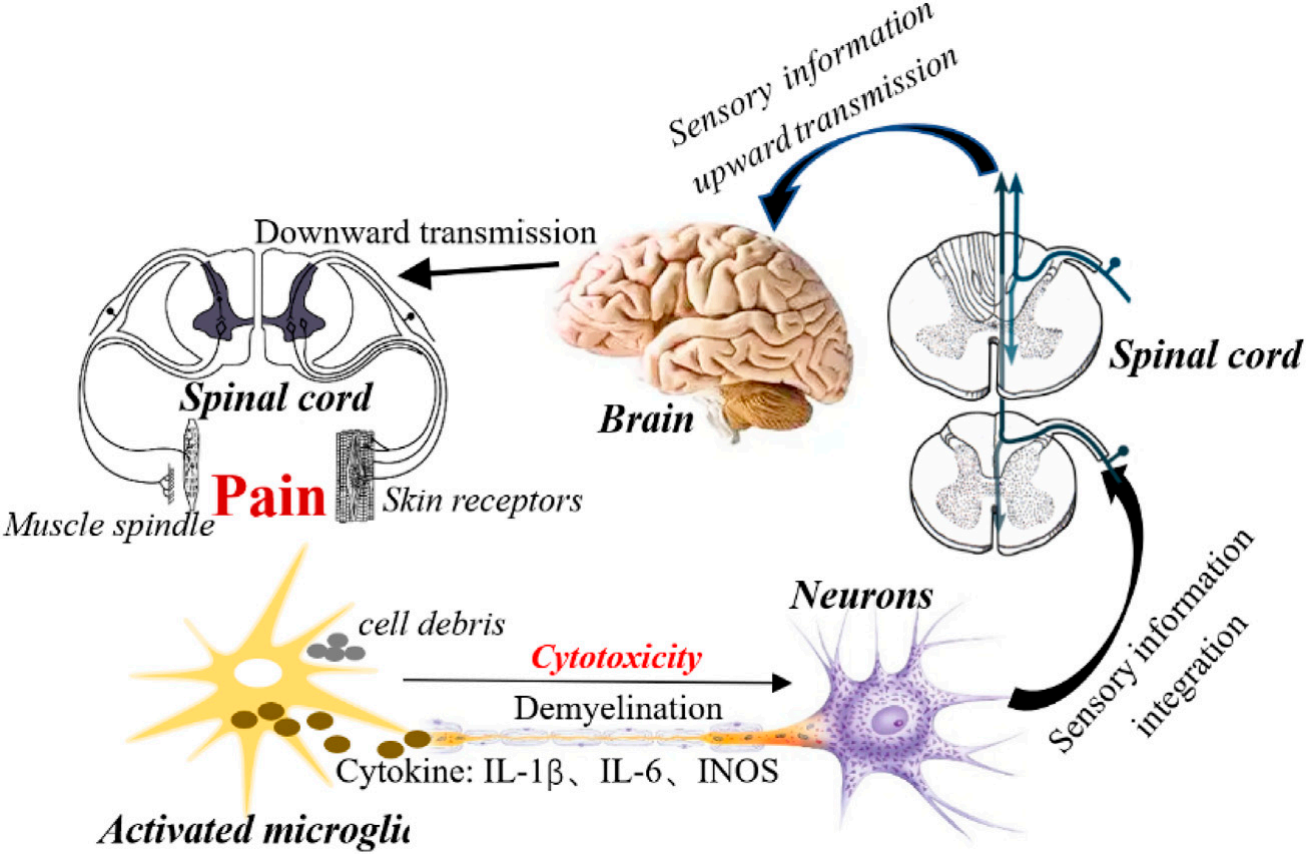
Activation of microglial is involved in the development of NPP. Activation of microglia increases the neuroinflammatory response around nerve injury by releasing a variety of cytotoxic factors (such as TNF-a, IL-1β, etc.). In addition, microglia activation transmits nociceptive information to neurons, enhances neuronal synaptic plasticity, enhances sensory information transmission, and stimulated neurons upload sensory information to the spinal cord center. Subsequently, the spinal cord center integrates sensory information to the higher center (brain) through the ascending conduction tract, and then the brain sends out instructions after receiving the information, and passes through the spinal cord through the descending conduction tract to the peripheral receptors (skin and muscle spindles), resulting in pain.

NPP is a symptom caused by direct or indirect damage to the nervous system, resulting in dysfunction or transient disturbance (Bouhassira, 2019). It is worth noting that pain can still exist or persist even after the elimination of nociceptive stimulation, which may be related to the transmission of neurological function and sensory information and the degree of nerve repair, as well as individual differences and psychological influences. Most of the pain is spontaneous pain and hyperalgesia (Meacham et al., 2017; Macone and Otis, 2018). Although the pathological mechanism of NPP is complex, which involves the participation of many factors. However, with the research on the pathological mechanism of NPP, some widespread recognitions have been obtained, which are reflected in the following aspects (Table 2):

a. Nociceptor sensitization and nociceptive fiber afferents (Sun et al., 2018; Simonetti and Kuner, 2020): Nerve damage can lead to decreased nerve fibers and demyelination of axons in the damaged area. It was found that the intracellular calcium and hyperexcitability of sensory neurons increased in the area of nerve injury, and the synaptic plasticity between afferent C fibers and dorsal horn neurons increased (Liu et al., 2015; Yang et al., 2017).
b. Microglia activation: Microglia are resident immune cells in the nervous system, widely distributed in the central nervous system, and are essential for maintaining the stability of the immune microenvironment of the nervous system (Voet et al., 2019). Although, under physiological conditions, microglia are in a quiescent state and lack immune and phagocytic functions. However, they still have a certain ability to migrate and play a certain protective role in the maintenance of the microenvironment in the brain and the stability of the nervous system function (Rajendran and Paolicelli, 2018). Indeed, microglia can migrate to the injury site and promptly remove cell metabolites and apoptotic cell debris, which is very important to ensure the relative stability of the brain environment and maintain the normal function of the central nervous system (Stadelmann et al., 2019; Abe et al., 2020). Moreover, activation of microglia also facilitates myelin formation and axonal regeneration after nerve injury (Pinto et al., 2021). Other studies reveal that efficient remyelination requires mediation of pro-inflammatory microglial death from proliferative to pro-regenerative states (Lloyd et al., 2019). This study implies that microglial cell death or impaired regeneration may underlie dysregulated microglial activation in neurological diseases. Additionally, microglial activation can induce pain by increasing neuroinflammatory responses. While inhibiting the activation of microglia prevents persistent stimulation of neuroinflammation and reduces pain (Inoue and Tsuda, 2018; Yi et al., 2021a; Navia-Pelaez et al., 2021). Studies have shown that 17β-estradiol inhibits the activation of microglia and astrocyte, reduces the expression of IL-1β, IL-6, iNOS and COX-2, and alleviates NPP caused by spinal cord injury (Lee et al., 2018b). Importantly, microglia can communicate with other glial cells and neurons, enhance the transmission of nociceptive information and increase pain perception (Jin et al., 2019; Zhou et al., 2019; Yi et al., 2021b) (Figure 2).
c. Abnormal neuronal activity (St John Smith, 2018; Vilela et al., 2021; Wercberger et al., 2021): Neurons stimulated by noxious factors can lead to abnormal discharge and enhanced synaptic plasticity, enhancing sensory information afferents (Terayama et al., 2015; Yang et al., 2021). In animal models of NPP, parabrachial neurons exhibit enhanced spontaneous and evoked activity and significantly increase afterdischarge responses (Raver et al., 2020). It is worth mentioning that the mutual communication between microglia and dorsal horn neurons also promotes the progression of pain. Indeed, activated microglia help spinal dorsal horn neurons to aggregate nociceptive input to induce NPP (Yamamoto et al., 2015; Terayama et al., 2018). Studies have shown that nerve injury is involved in P2Y12 receptor-dependent GTP-RhoA/ROCK2 signaling, increases the activation of microglia, and increases excitatory synaptic transmission in dorsal horn neurons (Yu et al., 2019), suggest that crosstalk between microglia and neurons is ultimately involved in the performance of nociceptive hyperalgesia. In addition, recent studies have found that ion channels, such as calcium channels, on nociceptor neurons help regulate nociceptive synaptic transmission (Chen et al., 2009; Ritter et al., 2015). For example, rapidly inactivating Kv3.4 potassium current dysfunction in neurons of dorsal root ganglion (DRG) is associated with persistent pain. (Zemel et al., 2017).
d. Neurotransmitter release (eg GABA) (Fan et al., 2022): Kv3.4 channel is a regulator of nerve cell excitability, which can change presynaptic membrane potential, affect glutamatergic fiber transmission, and generate pain afferents (Muqeem et al., 2018). For example, O-desmethyltramadol (M1) inhibits the quantum release of L-glutamate from nerve terminals by activating μ-opioid receptors, but not norepinephrine and serotonin receptors, inhibiting nociceptive information transmission (Koga et al., 2019). These primary afferents activate inhibitory interneurons of the spinal cord, which release (e.g., GABA and enkephalin) as transmitters to regulate pain inputs (Otsuka and Yanagisawa, 1990). Nociceptive receptor neurons release neuropeptides and neurotransmitters from nerve endings and regulate vascular, congenital and adaptive immune cell responses. These afferent fibers release neurotransmitters in the dorsal root ganglion and dorsal horn of the spinal cord and activate microglia as local immune cells. Activated microglia produce pro-inflammatory cytokines, chemokines and neuropeptides, which interact with secondary neurons to amplify the hypersensitivity of secondary neurons and trigger central sensitization (Vergne-Salle and Bertin, 2021).
e. Immune cell infiltration (such as macrophages, and T lymphocytes) (Durante et al., 2021; Micheli et al., 2021): Marked expansion and proliferation of macrophages around injured sensory neurons, involved in pain progression. Depletion of DRG macrophages reduces nerve injury-induced mechanical hypersensitivity and DRG macrophage expansion, prevents nerve injury-induced activation and proliferation of microglia, and reduces hyperalgesia (Yu et al., 2020). Agtr2 macrophages are the main immune cells that invade the site of nerve injury. Interestingly, neuropathic mechanical and cold hyperalgesia are mitigated by peripheral macrophage depletion (Shepherd et al., 2018a). Other studies show that angiotensin II triggers peripheral macrophage-sensory neuron redox crosstalk to trigger pain (Shepherd et al., 2018b).

**TABLE 2:** Potential mechanisms of neuropathic pain (NPP).

● Nociceptor sensitization and nociceptive fiber afferents
● Microglia activation
a. Immune function
b. Pro-inflammatory effects
c. Communication with neurons
● Abnormal neuronal activity: Abnormal discharge and enhanced synaptic plasticity
Ion channel inactivation
● Neurotransmitter release: Primary sensory afferent fibers release neurotransmitters in the dorsal root ganglion and dorsal horn of the spinal cord, activate microglia, promote inflammatory release, and amplify the hypersensitivity of secondary neurons
● Immune cell infiltration (macrophages): Expansion and proliferation
● Pro-inflammatory factor release

Taken together, the pathological mechanisms of NPP have received some consistent recognition, involving sensory neuron activity, microglia activation, inflammatory cell infiltration and pro-inflammatory factor release, enhanced neurotransmission, and central sensitization. This also means that inhibiting the activation of microglia, reducing the infiltration of inflammatory cells, protecting neurons, and improving the microenvironment of the nerve injury area may be potential mechanisms for the treatment of NPP. However, given that the pathological mechanism of NPP is complex and involves many factors, it is necessary to continuously explore and study the detailed mechanism of NPP.

## 5 The application of OECs in the treatment of NPP

The treatment of NPP is still a difficult problem to overcome at present. Usually, drugs are used to suppress pain or some physical rehabilitation therapy is used to relieve pain, but the effect is still not ideal, which also brings new challenges to researchers. Therefore, it is increasingly important to explore and find new treatments. Fortunately, in recent years, cell transplantation technology has entered people’s field of vision, and it has been well applied in the repair of nerve damage, and has also achieved satisfactory results. Although the effect in clinical trials is not very satisfactory, the exact role of cell transplantation in repairing damaged nerves and restoring function has been confirmed by different studies. Indeed, transplanting functional cells (such as neural stem cells and Schwann cells) into nerve injury has a good effect of suppressing pain (Zhang et al., 2018a; Du et al., 2019; Wang et al., 2021c). For example, Sertoli cells transplantation significantly improved the recovery of motor function and pain relief, which was related to the decrease of cavity, the expression level of TRPC6 and Caspase3 and the number of activated microglia after transplantation (Rahimi et al., 2022). In view of the unique biological characteristics of OECs, they have become candidate cells for the treatment of nerve injury. Correspondingly, it also exerts a better analgesic effect in pain treatment (Nakhjavan-Shahraki et al., 2018). Transplantation of OECs into spinal cord injury of rat found that the expression of NF200 was significantly increased, the expression of GFAP was decreased, and the sensory nerve function was improved (Zheng et al., 2017).

OECs play a role in pain relief by mediating the expression of some pain-related molecules. P2X purine receptors belong to ATP-dependent ion channel receptor, which is abundantly expressed in nervous system. For example, P2X2/3 receptor is expressed in sensory neurons, P2X4 receptor is expressed in microglia, while P2X7 receptor is expressed in neurons and microglia, and expressed in immune cells (Zhang, 2021). These purine receptors are highly expressed in pain and are closely related to the development of pain. An important mechanism of cell therapy is to exert the pharmacological characteristics of analgesia by reducing the expression of these P2X purine receptors. Transplantation of Schwann cells into sciatic nerve injury reduced the expression level of P2X2/3 receptor in the dorsal root ganglia and suppressed pain (Zhang et al., 2018b). OECs transplantation can relieve pain by down-regulating the expression of P2X purine receptors, which is also a well-studied pathological mechanism. OECs transplantation increased the expression of NF200, decreased the expression of GFAP, and inhibited the overexpression of P2X4 receptor, which plays an important role in NPP induced by spinal cord injury (Zheng et al., 2017). Our previous studies also found that OECs transplantation could significantly reduce the expression of P2X4 receptor in spinal cord and P2X2/3 receptor in dorsal root ganglion, and relieve NPP in rats (Zhao et al., 2015; Zhang et al., 2019b). Trigeminal neuralgia (TN) is a common facial nerve pain. OECs was transplanted into the ligation of infraorbital nerve in rats, the facial mechanical pain threshold increased significantly, which was related to the decreased expression of P2X7 receptor in trigeminal ganglion (Lu et al., 2022). In view of the fact that allogeneic cell transplantation will cause immune rejection, and OECs transplantation is likely to lead to the attack of inflammatory factors, resulting in a decrease in the survival rate of cells transplanted into the host, and the effect of pain relief is reduced. Therefore, later, we used the microencapsulation technology. Microcapsule is a lipid translucent membrane with good immune isolation, macromolecular substances are not easy to pass through, and it has the function of protecting transplanted cells (Olabisi, 2015; Mao et al., 2019; Santos et al., 2021). We transplanted microencapsulated OECs to the site of sciatic nerve injury and found significantly decreased the expression of P2X7 receptor in spinal cord and relieved neuropathic pain in rats. Interestingly, the effect of microencapsulated OECs on down-regulating the expression of P2X7 receptor and relieving pain was stronger than that of OECs alone (Zhang et al., 2019c). This also means that protecting the host OECs and reducing immune rejection may have a better biological function of pain relief, and a new model of cell therapy is proposed.

Under physiological conditions, the expression of NGF in primary sensory neurons was very low, but its expression level increased significantly after nerve injury. The study confirmed that NGF induced the transfer of TRPV1 to the membrane of neurons (Lawrence et al., 2021). However, these neurotrophic activities lead to axonal terminal budding and help to increase local pain sensitivity (Ro et al., 1999). In spinal cord injury, the transplanted neural stem cells restored the sensory function of rats with spinal cord injury, while OECs caused hyperalgesia. However, co-transplantation could promote the survival of neural stem cells and reverse the hyperalgesia induced by OECs. The mechanism may be related to the down-regulation of NGF (Luo et al., 2013). Brain-derived neurotrophic factor (BDNF) is an important member of the NGF family, and its high expression is involved in pain (such as neuropathic pain and trigeminal neuralgia). Studies have shown that transplantation of OECs into the hemi-transected spinal cord can lead to hyperalgesia, possibly due to upregulation of brain-derived neurotrophic factor (BDNF) (Lang et al., 2013). There are differences in the possible causes of different contradictions, the parts and causes of inducing pain, the dose and time of OECs transplantation, and the transplantation methods. In addition, an important point may be related to the regulation of the expression of pain-related molecules by OECs transplantation.

Another characteristic of OECs in nerve injury and pain relief is that activated OECs can effectively promote the proliferation and migration of vascular endothelial cells and the formation of vascular-like structures (Wang et al., 2022). After transplantation of OECs after photochemical injury of spinal cord in rats, the BBB score, motor and somatosensory evoked potential amplitudes were significantly increased, the expression of COX-2 and VEGF of astrocytes in spinal cord tissue increased, and the vascular density increased at the site of spinal cord injury (López-Vales et al., 2004).

The beneficial effect of OECs on pain is attributed to the ability of OECs to cross the boundary of PNS-CNS. The growth factors, cell adhesion molecules and extracellular matrix proteins they produce promote and guide axonal growth, and their ability to remyelinate axons. OECs are thought to help axon regeneration by removing necrotic cells (necrotic bodies) and myelin fragments (Wu et al., 2011). It was found that Schwann cells could produce a small amount of pro-inflammatory cytokine TNF-α, while OECs did not produce detectable TNF-α and engulfed myelin fragments. This also means that OECs have higher phagocytosis and transport capacity than Schwann cells, produce a lower number of pro-inflammatory cytokines, and play a better role in the repair of injured nerves (Nazareth et al., 2020). All these create the basic conditions for the reconstruction of nerve function and pain relief. Other studies have found that 1 week after C7 and C8 dorsal root injury, transplantation of OECs into the dorsal horn effectively alleviated the neuropathological disorders associated with dorsal root injury, including spontaneous pain behavior, tactile hypersensitivity and thermal hyperalgesia (Wu et al., 2011). However, interestingly, delayed OECs transplantation did not improve the sensory control of complex, goal-oriented skillful stretching and ladder walking, and no significant effect of transplanted OECs on injury-induced central recombination and afferent sprouting was found (Wu et al., 2011). This phenomenon may be explained by the antinociceptive effect mediated by OECs transplantation by altering other mechanisms such as inflammation and astrocyte proliferation.

In view of the feasibility of OECs transplantation in the treatment of pain. Later, researchers co-transplanted OECs with other functional cells (such as neural stem cells) or bioscaffolds to relieve pain (Luo et al., 2013; Zhang et al., 2020b). OECs combined with neural stem cells were transplanted into spinal cord injury of rats. It was found that the BBB score of rats increased significantly. And the BBB score increased rapidly at 2 weeks, while the BBB score increased slightly at 6 weeks. The recovery of limb function was better than that of single cell transplantation, and the neat arrangement of spinal cord cells was close to normal (Guo et al., 2015). Moreover, they also found that OECs combined with NSCs can better induce neural stem cells to differentiate into neurons (Guo et al., 2015). The study showed that compared with SCs transplantation, the axonal regeneration of sciatic nerve stump after OECs transplantation was 25% less, but the axonal regeneration of sciatic nerve stump after SCs/OECs combined transplantation was 28% more, which significantly restored the motor and sensory function of rats (You et al., 2011). We later combined OECs with chitosan biomaterials and found that the combined transplantation was more effective in relieving pain than single-cell transplantation (Zhang et al., 2020c).

OECs have made some achievements in the treatment of nerve injury in clinical patients, and their contribution to repairing damaged nerves and restoring some functions is also affirmed. However, there are few reports on the use of OECs in the treatment of patients with NPP clinical. Transplantation of fetal OECs into 17 patients with intractable chronic pain with spinal cord injury aged 6-309 months (mean 102.2 months). The degree of pain before and after 0.5-88 months was compared with the International Society of Neurorepair Spinal Cord injury rating scale (IANRSCIFRS). 0 represents extremely uncontrollable pain, one point represents severe pain that needs and responds to anesthetics, two points represents mild pain that responds to common painkillers, and three points represents no pain. The results showed that pain improved by an average of 1.2 points after OECs transplantation (Chen et al., 2020). Another report is: a 72-year-old stroke patient with right limb sensory and functional impairment and pain for 8 years, 4-month fetal-derived OECs were transplanted into the patient’s brain (cell volume 1x10^10^/L^-1^). The results showed that the patient had no adverse reactions such as fever, abnormal platelets and other complications after OECs transplantation (Liu et al., 2008). The pain of the right limb was reduced by 70% on the first day after the operation, 80% on the second day after the operation, and 90% on the third day after the operation. The muscle tension was reduced, and the function of the right limb recovered. The neurological function score increased significantly, and the patient was discharged from the hospital after 3 weeks (Lang et al., 2013). Although there are few reports in clinical trials, but OECs transplantation has a certain effect on pain relief and promotes functional recovery.

Although some data obtained from basic research support the feasibility of OECs for the treatment of pain, this is not enough to support extensive clinical development, which may require overcoming some difficulties. For example, there is still a lack of basic research data to support clinical development, the source and purity of OECs, the selection of cell transplantation time, the transplantation method and the control of the number of transplanted cells, immune rejection and the survival rate of cell transplantation, etc. Therefore, it is limited in clinical trials. However, the therapeutic effect of OECs transplantation on pain and nerve injury has been affirmed, so many problems need to be overcome in the future clinical treatment of OECs. Non-etheless, OECs hold promise as a promising new technology and method for promoting functional repair and pain relief.

## 6 Conclusion

Nerve injury is accompanied by sensory and functional disorders, leading to pain, which is a common symptom of most diseases. However, the treatment strategies for NPP are limited. Therefore, it is particularly important to find new treatment methods. Fortunately, the role of OECs in nerve injury repair has been affirmed and recognized by a large number of studies. Transplantation of OECs can promote regeneration of injured nerves and restore some body functions. Indeed, OECs transplantation can relieve pain, and exert analgesic effect for a longer period of time. The mechanism by which OECs exert their analgesic effect lies in their unique biological characteristics. Therefore, OECs are expected to be a promising method for the treatment of NPP and bringing new hope to patients in the future.

## Abbreviations

Neuropathic pain, NPP; Olfactory ensheathing cells, OECs; nerve stem cells, NSCs; dorsal root ganglion, DRG; entral nervous system, CNS; peripheral nervous system, PNS; spinal cord injury, SCI; interleukin-1β, IL-1β; NALP3, NACHT, LRR and PYD domains-containing protein 3; Tumor necrosis factor- α, TNF-α; interleukin-18, IL-18; Nitric oxide synthase, NOS; brain-derived neurotrophic factor, BDNF.
